# The association between hematocrit–albumin gap and sepsis risk in ICU patients: a multicenter cross-sectional study

**DOI:** 10.3389/fmed.2026.1747343

**Published:** 2026-04-17

**Authors:** Fenggao Zhou, Han Xia, Hongyin Zhu, Zhiyuan Xiao, Wangbin Xu, Yu Su

**Affiliations:** 1 Department of Internal Medicine Intensive Care Unit, The First People's Hospital of Yunnan Province, School of Medicine, Kunming University of Science and Technology, Kunming, Yunnan, China; 2 Department of Cardiology, The First Affiliated Hospital of Kunming Medical University, Kunming, Yunnan, China; 3 Department of Intensive Medicine, The First Affiliated Hospital of Kunming Medical University, Kunming, Yunnan, China

**Keywords:** critically ill patients, hematocrit-albumin gap, MIMIC-IV, non-linear association, sepsis

## Abstract

**Background:**

Sepsis is a major global health concern. Prior research has identified hematocrit and albumin levels as potential prognostic indicators for sepsis. Nevertheless, the specific relationship between the hematocrit–albumin gap (HAG) and susceptibility to sepsis remains underexplored. To address this study, we conducted a retrospective cross-sectional analysis using two independent datasets: (1) the MIMIC-IV database (derivation cohort) and (2) a retrospectively collected database from the Medical Intensive Care Unit (MICU) of The First People's Hospital of Yunnan Province, China (validation cohort).

**Objective:**

This study aims to elucidate the relationship between HAG and sepsis risk.

**Methods:**

This retrospective cross-sectional study utilized two independent datasets: (1) a derivation cohort of 19,134 adults with first-recorded ICU admissions during initial hospitalizations from the MIMIC-IV database and (2) an independent single-center validation cohort of 630 consecutive patients retrospectively enrolled from the Medical Intensive Care Unit (MICU) at The First People's Hospital of Yunnan Province, China (2022–2023). We collected data on hematocrit, albumin levels, and sepsis status. The study employed the logistic regression analysis and smooth curve fitting to analyze the data, with subgroup analyses conducted across different parameters and comorbidity statuses.

**Results:**

The investigation revealed an overall sepsis prevalence of 54.5%, disaggregated by gender: 56.1% among men and 43.9% among women. A multivariable-adjusted regression analysis determined the odds ratio (95% confidence interval) for sepsis—associated with HAG (per 7.848 increment)—to be 1.43 (95% CI: 1.37–1.48). Furthermore, a multivariate restricted cubic spline regression analysis revealed an S-shaped non-linear relationship between HAG and the incidence of sepsis, characterized by significant non-linearity (*P* < 0.05). When the HAG is below −9.162, the odds ratio (OR) for sepsis was 0.938, indicating a negative correlation. Between −9.162 and 17.27, a significant positive correlation exists with an OR of 1.051. Above 17.27, a relative saturation effect is observed, decreasing the OR to 1.023. Additionally, a significant statistical interaction was noted between HAG and conditions such as anemia, liver disease, and metastatic solid tumors in the prediction of sepsis (*P* < 0.05). Sensitivity analyses reinforced the reliability of the primary outcomes, supporting the study's conclusions.

**Conclusion:**

An S-shaped association was observed between the HAG and sepsis. Notably, the risk increased when the range of this difference was between −9.162 and 17.27. While HAG calculation is simple and rapid, its diagnostic utility requires prospective validation.

## Introduction

Sepsis, a critical condition characterized by life-threatening organ dysfunction resulting from a dysregulated host response to infection, poses a significant global health threat (1). It is estimated that approximately 31.5 million cases of sepsis occur globally each year, of which 19.4 million are severe, requiring hospitalization. These cases are associated with, or directly lead to, approximately 5.3 million deaths annually (2). Although the precise incidence of sepsis is difficult to pinpoint, conservative estimates highlight sepsis as a leading cause of death and critical illness across the world. The importance of early detection and intervention cannot be overstated, as they are crucial for significantly improving patient outcomes (3). The onset of sepsis is often insidious, and sepsis-induced organ dysfunction possibly may remain covert; unrecognized infections may underlie new-onset organ dysfunction (1). In clinical practice, patients frequently present with symptoms such as shock, abdominal pain, or respiratory distress, potentially indicative of preexisting sepsis, which may not be promptly diagnosed. Such delays in diagnosis can adversely impact the prognosis of sepsis.

The diagnosis of sepsis necessitates the support of hematological and biochemical biomarkers. Traditional biomarkers, such as procalcitonin (PCT), C-reactive protein (CRP), interleukin-6 (IL-6), lactate, and white blood cell count, often exhibit low sensitivity and specificity for sepsis identification, which complicates the diagnosis process. Additionally, some of these markers require prolonged analysis times, further impeding the rapid recognition and treatment of sepsis (4, 5). In addition, emerging biomarkers, such as lipopolysaccharide-binding protein (LBP), soluble triggering receptor expressed on myeloid cells-1 (sTREM-1), soluble urokinase plasminogen activator receptor (suPAR), and presepsin, face debates over their diagnostic value and are limited by high costs and low clinical adoption (4, 6).

Following the adoption of the Third International Consensus Definitions for Sepsis and Septic Shock (Sepsis-3)—which defines sepsis as life-threatening organ dysfunction arising from a dysregulated host response to infection (1)—timely identification of at-risk patients remains a persistent clinical challenge, particularly in resource-limited settings. The HAG, derived from two universally accessible, low-cost laboratory parameters, represents a pragmatic complementary indicator within the Sepsis-3 framework. Unlike the Systemic Inflammatory Response Syndrome (SIRS; limited specificity in critically ill populations (7)), the quick Sequential Organ Failure Assessment (qSOFA; suboptimal sensitivity in established ICU cohorts (8, 9)), or the full SOFA (requiring sequential multi-organ assessments (10)), hematocrit–albumin gap (HAG) can be readily computed within minutes of admission using routine blood tests. We propose a clinically actionable integration pathway: notable HAG elevations may prompt immediate SOFA assessment and targeted infection workup, thereby streamlining clinical workflows, accelerating time to recognition, and complementing Sepsis-3 diagnostics. Although prior studies have examined HAG in specific contexts (e.g., elderly cohorts (11) or patients with culture-proven infections (12)), notable limitations—including a narrow demographic scope, exclusion of culture-negative sepsis cases, and the application of pre-Sepsis-3 criteria—highlight the need for contemporary validation. Utilizing the Medical Information Mart for Intensive Care IV (MIMIC-IV) database (v2.2) and an independent validation cohort of medical intensive care unit (MICU) admissions from The First People's Hospital of Yunnan Province (2022–2023), this study aims to (1) rigorously evaluate the association between HAG and sepsis risk strictly defined by Sepsis-3 criteria and (2) examine whether the association between HAG and sepsis follows a non-linear pattern.

## Materials and methods

### Database

This study utilized two distinct data sources:

1. Derivation Database: Data were primarily extracted from the Medical Information Mart for Intensive Care-IV (MIMIC-IV v2.2), curated by the MIT Laboratory for Computational Physiology, and hosted on PhysioNet. This database contains de-identified clinical records from >50,000 ICU admissions at Beth Israel Deaconess Medical Center (2008–2019), including vital signs, laboratory measurements (hematocrit and albumin), medications, and outcomes.

2. Validation Database: To enhance generalizability, we retrospectively collected data from all consecutively admitted patients (*n* = 630) in the MICU at The First People's Hospital of Yunnan Province, China, between January 2022 and December 2023. This dataset included identical variables to the derivation set, with a diagnosis of sepsis adjudicated per Sepsis-3 criteria.

### Study population

In this study, we scrutinized the records of 73,181 patients admitted to the ICU from 2008 to 2019 in the MIMIC-IV database. Among these patients, 55,961 represented their first hospital admission. Our research centered on adult patients (18 years or older) admitted to the ICU for the first time. The exclusion criteria included the absence of hematocrit and albumin levels data on the first day of ICU admission. Concurrently, a validation cohort of 630 consecutive adults (≥18 years) meeting identical criteria was enrolled from the MICU at The First People's Hospital of Yunnan Province, China between 2022 and 2023.

### Exposure variable

The HAG was identified as the primary study variable. HAG is calculated by the difference between the initial hematocrit (%) and serum albumin (g/L) upon ICU admission. If there were multiple results for the above parameters within the first 24 h, the initial set of data was selected.

### Covariates

Confounding variables were selected from the MIMIC-IV database using a combination of clinical practice and references to previous scientific literature (11, 13, 14). Multicollinearity among these variables was evaluated using the variance inflation factor (VIF) method, with a VIF of ≥5 indicating the presence of multicollinearity.

The covariates included the following:

Demographic and admission conditions: age, gender, race, weight, and admission type.

Vital signs: temperature, heart rate, mean blood pressure (MBP), and respiration rate (RR).

Laboratory metrics: white blood cell (WBC), glucose, platelets, alanine aminotransferase (ALT), aspartate aminotransferase (AST), alkaline phosphatase (ALP), creatinine, blood urea nitrogen (BUN), lactate (Lac), anion gap (AG), and bicarbonate.

Medical history: congestive heart failure, chronic pulmonary disease, rheumatic disease, mild liver disease, severe liver disease, renal disease, diabetes, malignant cancer, and metastatic solid tumor.

### Anemia

Anemia was classified based on the World Health Organization's guidelines, which define it as a hemoglobin concentration below 13.0 g/dL in men and below 12.0 g/dL in women.

### Outcomes

The outcome of interest was whether patients were diagnosed with sepsis according to the Third International Consensus Definitions for Sepsis and Septic Shock (Sepsis-3) (1). Specifically, sepsis was defined as a life-threatening organ dysfunction caused by a dysregulated host response to infection, operationalized as an increase of ≥2 points in the Sequential Organ Failure Assessment (SOFA) score concurrent with suspected or documented infection. The calculation for the SOFA score in the MIMIC-IV dataset was performed using code from the MIT Laboratory for Computational Physiology, adhering to the SOFA criteria outlined in prior studies (15).

### Statistical analysis

The Kolmogorov–Smirnov test was used to assess the normality of the data. Continuous variables with normal distributions were described as mean ± SD, and those with skewed distributions were described as median (IQR). Categorical variables were summarized as frequencies (percentages). The independent samples Student's *t*-test or Mann–Whitney *U*-test evaluated continuous variables' differences across groups, based on their distribution. Chi-square or Fisher's exact tests analyzed categorical data differences.

We examined the influence of HAG on sepsis using a binary logistic regression analysis, yielding odds ratios (OR) and 95% confidence intervals (CIs), adjusting for relevant covariates. HAG was analyzed both as a categorical variable (five quantiles) and as a continuous variable (calculated per standard deviation, SD). Three models were constructed: Model 1 was adjusted for demographic variables and vital signs; Model 2 was adjusted for laboratory metrics and temperature; and Model 3, our primary model, further considered medical history.

To explore the HAG and sepsis non-linear dose-response, we employed a restricted cubic spline model with four knots, following Harrell's recommendations. Non-linearity was tested by integrating a quadratic term into our models. A two-piecewise regression model was used to determine the HAG's threshold effect on sepsis, with a smoothing plot indicating the breakpoint. We validated the continuous variable results and the threshold effect through a *P-value* for trend calculation after categorizing the HAG at this breakpoint. Subgroup analyses were preceded by categorizing continuous variables by clinical thresholds for interaction tests. Missing data were addressed through multiple imputation (five replications) using a chained equation method, optimizing statistical power, and reducing bias. A sensitivity analysis, including patients with various chronic conditions, evaluated the robustness of our findings and the impact of different models on the conclusions, with all effect sizes and *P*-*values* documented and analyzed. All analyses were performed using R Statistical Software (Version 4.2.2, http://www.R-project.org, The R Foundation) and the Free Statistics analysis platform (Version 1.9, Beijing, China, http://www.clinicalscientists.cn/freestatistics). A two-sided *P-value* of less than 0.05 was considered statistically significant.

## Results

### The selection of participants

**Figure 1:**
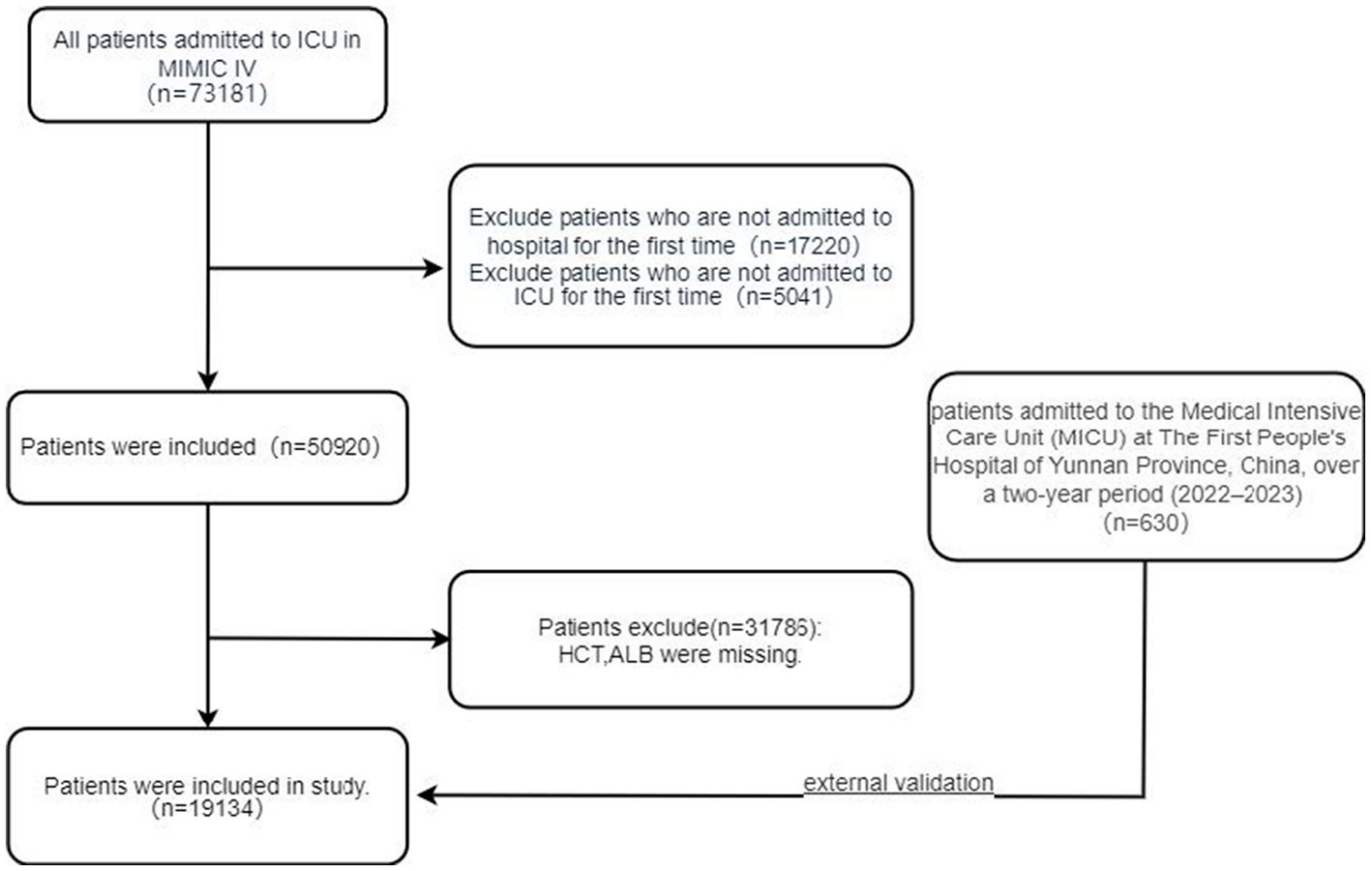
Flowchart of study patient enrollment.

The MIMIC-IV database contained 73,181 patients with ICU admissions. We identified 50,920 patients who were admitted to the hospital for the first time and were initially admitted to the ICU. After excluding 31,786 patients with missing HCT and ALB values, 19,134 patients were included in the derivation cohort. For validation, we retrospectively enrolled 630 consecutive patients meeting identical inclusion and exclusion criteria from the Medical Intensive Care Unit (MICU) of The First People's Hospital of Yunnan Province, China (2022–2023). The participant flow is illustrated in Figure 1.

### Baseline characteristics of participants

The study included 19,134 patients with an average age of 63.8 ± 17.7 years; 43.9% were female, and 56.1% were male. Table 1a presents the general characteristics of participants by the HAG group. Significant differences were observed across HAG groups in terms of sex, age, race, weight, admission type, vital signs, and laboratory metrics, as well as in the prevalence of congestive heart failure, renal disease, diabetes, liver disease, and metastatic solid tumor mellitus, with all comparisons yielding *P-values* less than 0.05. In contrast, the distribution of other patient characteristics, including chronic pulmonary disease, rheumatic disease, and malignant cancer, showed no significant differences among the HAG groups, as indicated by *P*-*values* greater than 0.05. Baseline characteristics of the validation cohort are presented separately in Table 1b.

**Table 1a:** Baseline characteristics of participants and outcome parameters in the derivation cohort.

Variables	Total	HAG (Quintile)					*P-value*
	*n* = 19,134	Q1 (*n* = 3,753)	Q2 (*n* = 3,836)	Q3 (*n* = 3,783)	Q4 (*n* = 3,902)	Q5 (*n* = 3,860)	
		(−29.4 to −3.6)	(−3.5 to 0.4)	(0.5 to 3.9)	(4.0 to 8.7)	(8.8 to 43.8)	
Sepsis, *n* (%)	10,429 (54.5)	1,571 (41.9)	1,758 (45.8)	1,949 (51.5)	2,358 (60.4)	2,793 (72.4)	< 0.001
Age, year	63.8 ± 17.7	63.3 ± 18.2	64.0 ± 18.4	63.9 ± 17.8	64.3 ± 17.0	63.4 ± 17.1	0.083
Gender, *n* (%)							< 0.001
Female	8,399 (43.9)	1,823 (48.6)	1,774 (46.2)	1,671 (44.2)	1,610 (41.3)	15,21 (39.4)	
Male	10,735 (56.1)	1,930 (51.4)	2,062 (53.8)	2,112 (55.8)	2,292 (58.7)	2,339 (60.6)	
Race, *n* (%)							< 0.001
Other	7,043 (36.8)	1,362 (36.3)	1,342 (35)	1,349 (35.7)	1,463 (37.5)	1,527 (39.6)	
White	12,091 (63.2)	2,391 (63.7)	2,494 (65)	2,434 (64.3)	2,439 (62.5)	2,333 (60.4)	
Weight (kg)	81.1 ± 27.1	78.9 ± 21.8	79.8 ± 22.4	81.4 ± 26.8	81.8 ± 24.6	83.4 ± 36.7	< 0.001
Admission type, *n* (%)							< 0.001
None EM	3,693 (19.3)	724 (19.3)	757 (19.7)	793 (21)	733 (18.8)	686 (17.8)	
EMER	15,441 (80.7)	3,029 (80.7)	3,079 (80.3)	2,990 (79)	3,169 (81.2)	3,174 (82.2)	
Vital signs							
Temperature, °C	37.4 ± 0.8	37.3 ± 0.7	37.4 ± 0.7	37.4 ± 0.8	37.4 ± 0.8	37.4 ± 1.0	< 0.001
Heart rate, bpm	105.6 ± 21.8	101.8 ± 20.0	102.5 ± 20.4	103.7 ± 21.5	106.8 ± 21.9	113.0 ± 22.9	< 0.001
MBP, mmHg	59.7 ± 15.0	61.1 ± 14.1	61.6 ± 14.1	60.9 ± 14.8	59.1 ± 15.0	56.0 ± 16.4	< 0.001
RR, bpm	28.3 ± 6.7	27.6 ± 6.4	27.6 ± 6.4	28.0 ± 6.5	28.7 ± 6.7	29.5 ± 7.1	< 0.001
Laboratory metrics							
WBC, K/uL	12.2 (8.5, 17.2)	10.5 (7.5, 14.5)	11.0 (8.1, 15.4)	12.0 (8.6, 16.6)	13.0 (9.2, 18.4)	15.2 (10.3, 21.2)	< 0.001
Hemoglobin, g/dL	10.3 ± 2.4	9.1 ± 2.2	10.2 ± 2.2	10.5 ± 2.3	10.6 ± 2.3	11.0 ± 2.5	< 0.001
Platelets, K/uL	174.0 (116.0, 236.0)	178.0 (116.0, 238.0)	183.0 (129.0, 242.0)	183.0 (128.0, 242.0)	171.0 (114.0, 237.5)	154.0 (98.0, 220.0)	< 0.001
Glucose, mg/dL	146.0 (116.0, 199.0)	141.0 (114.0, 185.0)	140.0 (113.0, 183.0)	142.0 (113.0, 190.0)	147.0 (117.0, 203.0)	162.0 (125.0, 233.0)	< 0.001
ALT, IU/L	28.0 (17.0, 64.0)	23.0 (15.0, 42.0)	25.0 (16.0, 48.0)	28.0 (16.0, 58.0)	31.0 (18.0, 79.0)	43.0 (21.0, 134.0)	< 0.001
AST, IU/L	41.0 (24.0, 102.0)	33.0 (22.0, 64.0)	34.0 (22.0, 70.0)	39.0 (23.0, 88.0)	45.0 (26.0, 119.0)	68.0 (32.0, 227.0)	< 0.001
ALP, IU/L	83.0 (62.0, 122.0)	79.0 (61.0, 110.0)	80.0 (61.0, 112.0)	83.0 (63.0, 120.0)	86.0 (63.0, 131.2)	90.0 (63.0, 141.0)	< 0.001
Creatinine, mg/dL	1.1 (0.8, 1.8)	1.1 (0.8, 2.0)	1.0 (0.8, 1.5)	1.0 (0.8, 1.6)	1.1 (0.8, 1.7)	1.3 (0.9, 2.1)	< 0.001
BUN, mg/dL	22.0 (15.0, 38.0)	23.0 (15.0, 44.0)	20.0 (14.0, 34.0)	20.0 (14.0, 33.0)	22.0 (15.0, 37.0)	26.0 (16.0, 42.0)	< 0.001
Lac, mmol/L	2.3 (1.5, 3.9)	2.0 (1.3, 3.1)	2.0 (1.4, 3.2)	2.1 (1.4, 3.4)	2.4 (1.6, 4.0)	3.2 (2.0, 5.8)	< 0.001
AG, mEq/L	17.7 ± 5.6	18.0 ± 5.4	17.0 ± 4.9	17.0 ± 5.3	17.4 ± 5.5	19.0 ± 6.6	< 0.001
Bicarbonate, mEq/L	20.7 ± 5.2	21.4 ± 5.0	21.5 ± 4.7	21.3 ± 4.9	20.6 ± 5.1	18.7 ± 5.7	< 0.001
Medical history							
CHF, *n* (%)	4,688 (24.5)	963 (25.7)	884 (23)	885 (23.4)	1,003 (25.7)	953 (24.7)	0.013
CPD, *n* (%)	4,327 (22.6)	813 (21.7)	837 (21.8)	843 (22.3)	916 (23.5)	918 (23.8)	0.084
Rheumatic disease, *n* (%)	615 (3.2)	123 (3.3)	120 (3.1)	105 (2.8)	139 (3.6)	128 (3.3)	0.389
Liver disease, *n* (%)	3,728 (19.5)	725 (19.3)	574 (15)	636 (16.8)	800 (20.5)	993 (25.7)	< 0.001
Renal disease, *n* (%)	3,684 (19.3)	985 (26.2)	728 (19)	676 (17.9)	680 (17.4)	615 (15.9)	< 0.001
Diabetes, *n* (%)	5,341 (27.9)	1,150 (30.6)	1,037 (27)	1,083 (28.6)	1,062 (27.2)	1,009 (26.1)	< 0.001
Malignant cancer, *n* (%)	2,777 (14.5)	528 (14.1)	533 (13.9)	515 (13.6)	578 (14.8)	623 (16.1)	0.012
Metastatic solid tumor, *n* (%)	1,338 (7.0)	195 (5.2)	273 (7.1)	255 (6.7)	278 (7.1)	337 (8.7)	< 0.001

HAG, hematocrit–albumin gap; EM, emergency; MBP, mean blood pressure; RR, respiration rate; WBC, white blood cell; ALT, alanine aminotransferase; AST, aspartate aminotransferase; ALP, alkaline phosphatase; BUN, blood urea nitrogen; Lac, lactate; AG, anion gap; CHF, congestive heart failure; CPD, chronic pulmonary disease.

**Table 1b:** Baseline characteristics of participants and outcome parameters in the validation cohort.

Variables	Total	HAG(Quintile)					*P-value*
	*n* = 630	Q1 (*n* = 126)	Q2 (*n* =125)	Q3 (*n* = 127)	Q4 (*n* = 126)	Q5 (*n* = 126)	
		(−26.5 to −3.9)	(−3.8 to 0.5)	(0.6 to 5.0)	(5.1 to 10.7)	(10.8 to 38.5)	
Sepsis, *n* (%)	299 (47.5)	28 (22.2)	46 (36.8)	40 (31.5)	89 (70.6)	96 (76.2)	< 0.001
Age, year	63.6 ± 16.0	59.9 ± 17.7	64.1 ± 15.6	61.7 ± 16.6	66.0 ± 15.5	66.3 ± 13.5	0.004
Gender, *n* (%)							< 0.001
Female	430 (68.3)	73 (57.9)	77 (61.6)	82 (64.6)	97 (77)	101 (80.2)	
Male	200 (31.7)	53 (42.1)	48 (38.4)	45 (35.4)	29 (23)	25 (19.8)	
Race, *n* (%)							
Other	630 (100.0)	126 (100)	125 (100)	127 (100)	126 (100)	126 (100)	
White	0 (0.0)						
Weight (kg)	61.9 ± 13.5	57.0 ± 11.2	57.9 ± 12.9	60.8 ± 13.1	65.4 ± 14.6	68.3 ± 11.9	< 0.001
Admission type, *n* (%)							0.005
None EM	423 (67.1)	69 (54.8)	80 (64)	90 (70.9)	89 (70.6)	95 (75.4)	
EMER	207 (32.9)	57 (45.2)	45 (36)	37 (29.1)	37 (29.4)	31 (24.6)	
Vital signs							
Temperature, °C	36.9 ± 0.9	36.9 ± 0.9	36.9 ± 0.9	37.0 ± 1.0	36.8 ± 0.8	36.9 ± 1.0	0.789
Heart rate, bpm	98.2 ± 22.5	97.3 ± 21.0	96.0 ± 20.5	95.6 ± 19.8	98.5 ± 22.8	103.4 ± 27.2	0.042
MBP, mmHg	90.2 ± 21.0	92.5 ± 20.0	91.4 ± 22.0	87.8 ± 22.0	89.9 ± 20.2	89.3 ± 20.5	0.43
RR, bpm	18.6 ± 6.5	18.3 ± 6.3	18.2 ± 6.7	19.0 ± 7.1	17.6 ± 5.2	19.7 ± 7.0	0.099
Laboratory metrics							
WBC, K/uL	10.4 (7.0, 15.1)	9.0 (6.6, 14.9)	10.3 (7.1, 13.8)	10.7 (6.9, 15.0)	11.0 (7.6, 15.0)	10.5 (7.4, 15.7)	0.45
Hemoglobin, g/dL	109.1 ± 33.1	73.0 ± 20.4	92.6 ± 23.2	110.2 ± 22.7	123.9 ± 20.8	145.6 ± 21.5	< 0.001
Platelets, K/uL	164.5 (103.0, 246.5)	181.5 (105.2, 266.2)	169.0 (99.0, 242.0)	178.0 (110.5, 255.5)	166.5 (115.0, 239.5)	138.0 (76.0, 198.5)	0.023
Glucose, mg/dL	8.1 (6.1, 11.2)	7.3 (5.6, 9.6)	7.8 (6.2, 10.1)	8.0 (6.0, 11.2)	9.5 (6.6, 13.7)	8.9 (6.2, 12.6)	< 0.001
ALT, IU/L	27.4 (15.1, 55.4)	20.4 (10.4, 37.5)	22.4 (11.7, 42.0)	29.1 (16.5, 52.0)	32.8 (19.4, 80.8)	34.0 (18.5, 72.5)	< 0.001
AST, IU/L	36.0 (22.0, 76.0)	28.0 (19.0, 52.8)	33.0 (21.0, 56.0)	36.0 (25.0, 70.0)	42.0 (24.0, 100.5)	51.0 (24.2, 120.0)	< 0.001
ALP, IU/L	85.0 (62.0, 119.0)	74.5 (56.0, 121.1)	82.0 (57.0, 113.0)	84.0 (67.0, 118.5)	86.5 (65.5, 121.9)	87.5 (64.5, 121.2)	0.129
Creatinine, mg/dL	91.0 (59.0, 228.8)	119.5 (53.5, 438.0)	83.0 (53.0, 293.0)	89.0 (58.0, 191.0)	92.0 (62.0, 149.5)	86.0 (65.2, 169.2)	0.256
BUN, mg/dL	10.5 (6.1, 18.3)	12.7 (6.9, 23.1)	11.0 (5.1, 19.1)	8.2 (5.6, 15.4)	9.3 (5.8, 15.5)	10.9 (7.0, 15.5)	0.006
Lac, mmol/L	1.6 (1.1, 2.7)	1.3 (0.9, 2.4)	1.5 (1.0, 2.4)	1.6 (1.1, 2.7)	1.7 (1.1, 2.6)	2.1 (1.5, 3.4)	< 0.001
AG, mEq/L	11.0 (8.0, 14.0)	12.0 (9.0, 15.0)	11.0 (9.0, 15.0)	11.0 (8.0, 14.0)	10.0 (8.0, 13.0)	10.0 (7.0, 14.0)	0.037
Bicarbonate, mEq/L	20.2 ± 6.1	20.1 ± 6.6	19.5 ± 5.9	20.4 ± 5.5	20.3 ± 5.3	20.7 ± 6.9	0.566
Medical history							
CHF, *n* (%)	121 (19.2)	27 (21.4)	19 (15.2)	25 (19.7)	19 (15.1)	31 (24.6)	0.243
CPD, *n* (%)	212 (33.7)	37 (29.4)	38 (30.4)	31 (24.4)	47 (37.3)	59 (46.8)	0.002
Rheumatic disease, *n* (%)	96 (15.2)	27 (21.4)	23 (18.4)	19 (15)	15 (11.9)	12 (9.5)	0.062
Liver disease, *n* (%)	59 (9.4)	14 (11.1)	8 (6.4)	13 (10.2)	8 (6.3)	16 (12.7)	0.302
Renal disease, *n* (%)	94 (14.9)	36 (28.6)	24 (19.2)	12 (9.4)	12 (9.5)	10 (7.9)	< 0.001
Diabetes, *n* (%)	172 (27.3)	30 (23.8)	37 (29.6)	39 (30.7)	34 (27)	32 (25.4)	0.72
Malignant cancer, *n* (%)	70 (11.1)	12 (9.5)	20 (16)	16 (12.6)	10 (7.9)	12 (9.5)	0.264
Metastatic solid tumor, *n* (%)	19 (3.0)	4 (3.2)	5 (4)	6 (4.7)	1 (0.8)	3 (2.4)	0.381

HAG, hematocrit–albumin gap; EM, emergency; MBP, mean blood pressure; RR, respiration rate; WBC, white blood cell; ALT, alanine aminotransferase; AST, aspartate aminotransferase; ALP, alkaline phosphatase; BUN, blood urea nitrogen; Lac, lactate; AG, anion gap; CHF, congestive heart failure; CPD, chronic pulmonary disease.

### Outcomes

The overall sepsis prevalence was 54.5% in the derivation cohort and 47.5% in the validation cohort. Prevalence by HAG quintiles showed:

Derivation cohort: Q1 = 41.9%, Q2 = 45.8%, Q3 = 51.5%, Q4 = 60.4%, Q5 = 72.4%

Validation cohort: Q1 = 22.2%, Q2 = 36.8%, Q3 = 31.5%, Q4 = 70.6%, Q5 = 76.2%

### Univariate analysis of factors related to sepsis

A univariate logistic regression analysis (Supplementary File 1a) revealed that HAG, age, gender, admission type, temperature, heart rate, mean blood pressure (MBP), respiratory rate (RR), white blood cell count (WBC), glucose, platelets, alanine aminotransferase (ALT), aspartate aminotransferase (AST), alkaline phosphatase (ALP), creatinine, blood urea nitrogen (BUN), lactate (Lac), bicarbonate, congestive heart failure, chronic pulmonary disease, rheumatic disease, liver disease, renal disease, diabetes, and malignant cancer were factors related to the incidence of sepsis. Women have a lower risk of developing sepsis than men. MBP and bicarbonate were negatively correlated with the occurrence of sepsis, indicating a protective effect against the condition. The remaining factors were positively correlated with the risk of developing sepsis, suggesting an increased risk associated with these factors. Corresponding univariate analyses for the validation cohort are presented in Supplementary File 1b.

### HAG and sepsis

In multivariable logistic regression analyses, after adjusting for potential confounders as shown in Table 2a, Model 3, the HAG, when treated as a continuous variable (per SD), was positively associated with the probability of sepsis (OR = 1.43, 95% CI = 1.37–1.48, *P* < 0.001). Furthermore, the analyses revealed that the risk of sepsis was 3.53 times higher in the Q5 group than in the Q1 group (OR = 3.53, 95% CI = 1.99–6.26, *P* < 0.001). Interestingly, participants in the Q2 group experienced a slight reduction in risk than Q1 (OR = 0.95, 95% CI = 0.66–1.37), suggesting a non-linear relationship in the derivation cohort.

**Table 2a:** Weighted odds ratios (95% confidence intervals) of HAG and sepsis in different models (derivation cohort).

Variable	Non-adjusted	Before multiple interpolation			After multiple interpolation		
		Model 1	Model 2	Model 3	Model 1	Model 2	Model 3
HCT–ALB per SD	1.58 (1.53–1.63)^*^	1.47 (1.42–1.52)^*^	1.35 (1.29–1.41)^*^	1.36 (1.3–1.42)^*^	1.47 (1.42–1.52)^*^	1.42 (1.37–1.48)^*^	1.43 (1.37–1.48)^*^
HCT–ALB (Quintile)							
Q1 (−29.4 to −3.6)	Ref	Ref	Ref	Ref	Ref	Ref	Ref
Q2 (−3.5 to 0.4)	1.18 (1.07–1.29)^*^	1.21 (1.1–1.34)^*^	1.39 (1.22–1.58)^*^	1.42 (1.25–1.61)^*^	1.19 (1.08 −1.31)^*^	1.34 (1.21– 1.48)^*^	1.38 (1.24–1.52)^*^
Q3 (0.5 to 3.9)	1.48 (1.35–1.62)^*^	1.46 (1.33 −1.62)^*^	1.59 (1.4–1.8)^*^	1.62 (1.42–1.84)^*^	1.44 (1.31–1.59)^*^	1.61 (1.45–1.78)^*^	1.65 (1.49–1.83)^*^
Q4 (4.0 to 8.7)	2.12 (1.94–2.32) ^*^	1.9 (1.72–2.1)^*^	1.81 (1.6–2.06)^*^	1.83 (1.61–2.08)^*^	1.92 (1.74–2.11)^*^	1.99 (1.79–2.2)^*^	2.01 (1.81–2.23)^*^
Q5 (8.8 to 43.8)	3.64 (3.3–4)^*^	3.08 (2.77–3.42)^*^	2.39 (2.09 to 2.73)^*^	2.4 (2.1–2.75)^*^	3.03 (2.74–3.36)^*^	2.67 (2.39–2.98)^*^	2.69 (2.41–3.01)^*^
*P* for trend	< 0.001	< 0.001	< 0.001	< 0.001	< 0.001	< 0.001	< 0.001

^*^P-value < 0.001.

Correspondingly, in the validation cohort (Table 2b, Model 3), the HAG as a continuous variable (per SD) was positively associated with sepsis (OR = 3.30, 95% CI = 2.30–4.72). The risk gradient was significantly amplified: Q5 vs. Q1 showed 31.20-fold higher risk (OR = 31.20, 95% CI = 11.14–87.41), with sequentially increasing odds from Q2 (OR = 2.70, 95% CI = 1.39–5.24) to Q4 (OR = 21.58, 95% CI = 9.08–51.30) also suggesting a non-linear relationship between HAG and sepsis risk (Table 2b, Model 3).

**Table 2b:** Weighted odds ratios (95% confidence intervals) of HAG and sepsis in different models (validation cohort).

Variable	Non-adjusted	Model 1	Model 2	Model 3
HCT–ALB per SD	2.5 (2.04–3.06)^*^	2.4 (1.94–2.97)^*^	3.18 (2.24–4.51)^*^	3.3 (2.3–4.72)^*^
HCT–ALB (Quintile)				
Q1 (−26.5 to −3.9)	Ref	Ref	Ref	Ref
Q2 (−3.8 to 0.5)	2.01 (1.15–3.53)^*^	2.01 (1.15–3.53)^*^	2.73 (1.43–5.23)^*^	2.7 (1.39–5.24)^*^
Q3 (0.6 to 5.0)	1.61 (0.91–2.86)	1.61 (0.91–2.86)	2.44 (1.17–5.11)^*^	2.64 (1.24–5.6)^*^
Q4 (5.1 to 10.7)	8.01 (4.43–14.47)^*^	8.01 (4.43–14.47)^*^	19.26 (8.31–44.61)^*^	21.58 (9.08–51.3)^*^
Q5 (10.8 to 38.5)	10.55 (5.66–19.65)^*^	10.55 (5.66–19.65)^*^	24.37 (9.02–65.79)^*^	31.2 (11.14–87.41)^*^
*P* for trend	< 0.001	< 0.001	< 0.001	< 0.001

Model 1 was adjusted for demographic and admission conditions (age, gender, race, weight, and admission type) + vital signs (temperature, heart rate, MBP, and RR);

Model 2 was additionally adjusted for laboratory metrics (WBC, glucose, platelets, ALT, AST, ALP, creatinine, bun, lac, AG, and bicarbonate);

Model 3 was additionally adjusted for medical history (congestive heart failure, chronic pulmonary disease, rheumatic disease, liver disease, renal disease, diabetes, malignant cancer, and metastatic solid tumor).

^*^P-value < 0.001.

HAG, hematocrit–albumin gap; EM, emergency; MBP, mean blood pressure; RR, respiration rate; WBC, white blood cell; ALT, alanine aminotransferase; AST, aspartate aminotransferase; ALP, alkaline phosphatase; BUN, blood urea nitrogen; Lac, lactate; AG, anion gap.

### The non-linear relationship between HAG and sepsis

**Figure 2:**
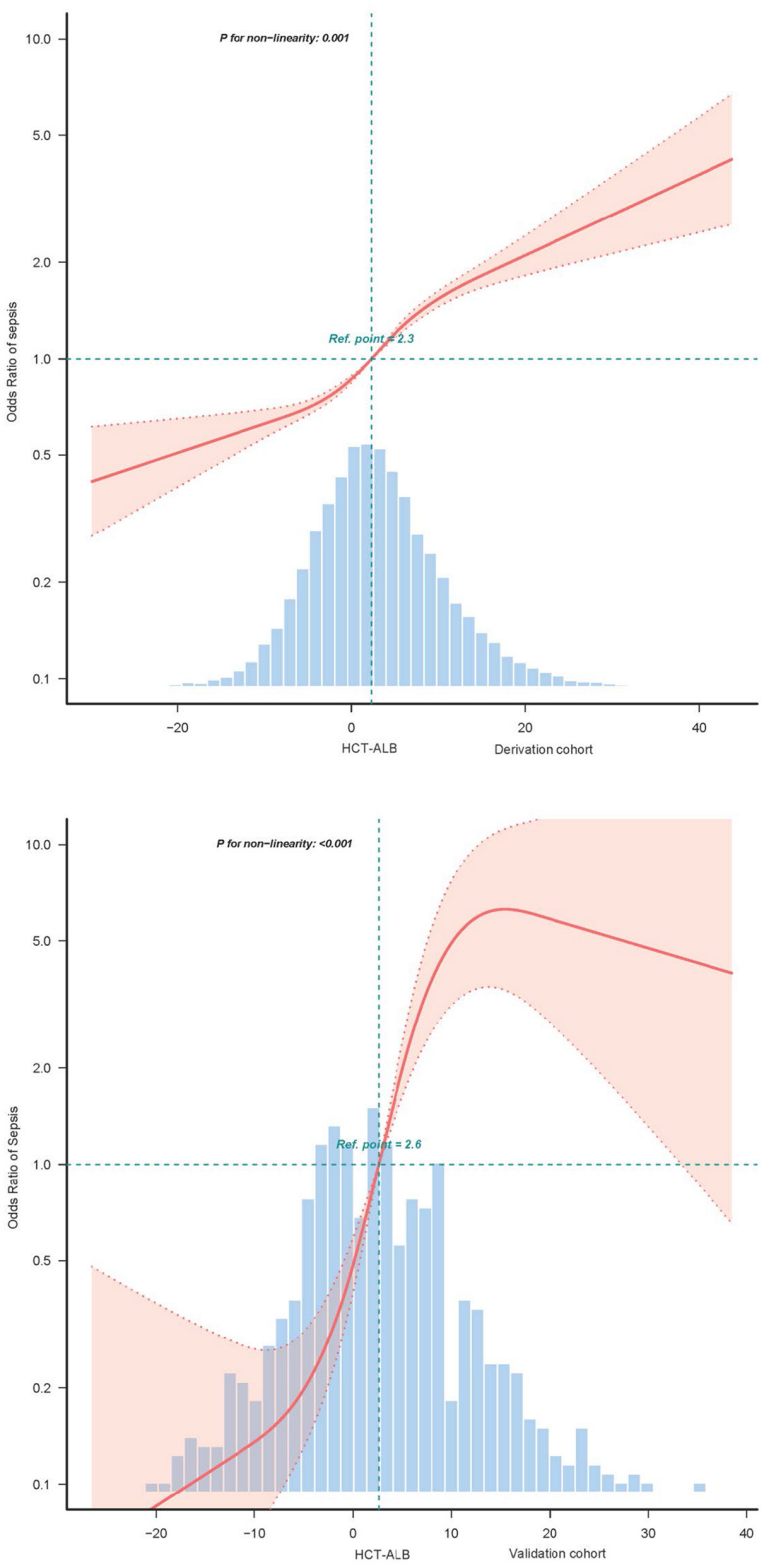
S-shaped non-linear dose–response relationship between HAG and sepsis. Data were fit by a multivariable logistic regression model based on restricted cubic splines. HAG was entered as continuous variable. Data were adjusted for demographic and admission conditions + vital signs + laboratory metrics + medical history. Here, the median HAG was defined as the reference standard. The red area represents the 95% CI.

The study utilized restricted cubic splines analysis, confirming a non-linear (S-shaped) relationship between HAG levels and sepsis risk in both cohorts (Derivation: *P* for non-linearity = 0.001, Validation: *P* < 0.001; Figure 2). Through a two-piecewise linear regression model, two inflection points at HAG levels of −9.162 and 17.27 were identified. Below −9.162, sepsis risk decreased with higher HAG (OR = 0.938, 95% CI = 0.894–0.985). Between −9.162 and 17.27, a positive correlation between HAG and sepsis risk was observed (OR = 1.051, 95% CI = 1.044–1.057). Above 17.27, the risk was slightly increased but not significantly (OR = 1.023, 95% CI = 0.97–1.079) (Table 3).

**Table 3:** Threshold effect analysis of the relationship between HAG and sepsis.

HAG	OR (95%CI)	*P-value*
< −9.162	0.938 (0.894–0.985)	0.0096
−9.162 to 17.27	1.051 (1.044–1.057)	< 0.001
>17.27	1.023 (0.97–1.079)	0.3922
Likelihood Ratio test		< 0.001

HAG, hematocrit-albumin gap.

### Sensitivity analysis

**Figure 3:**
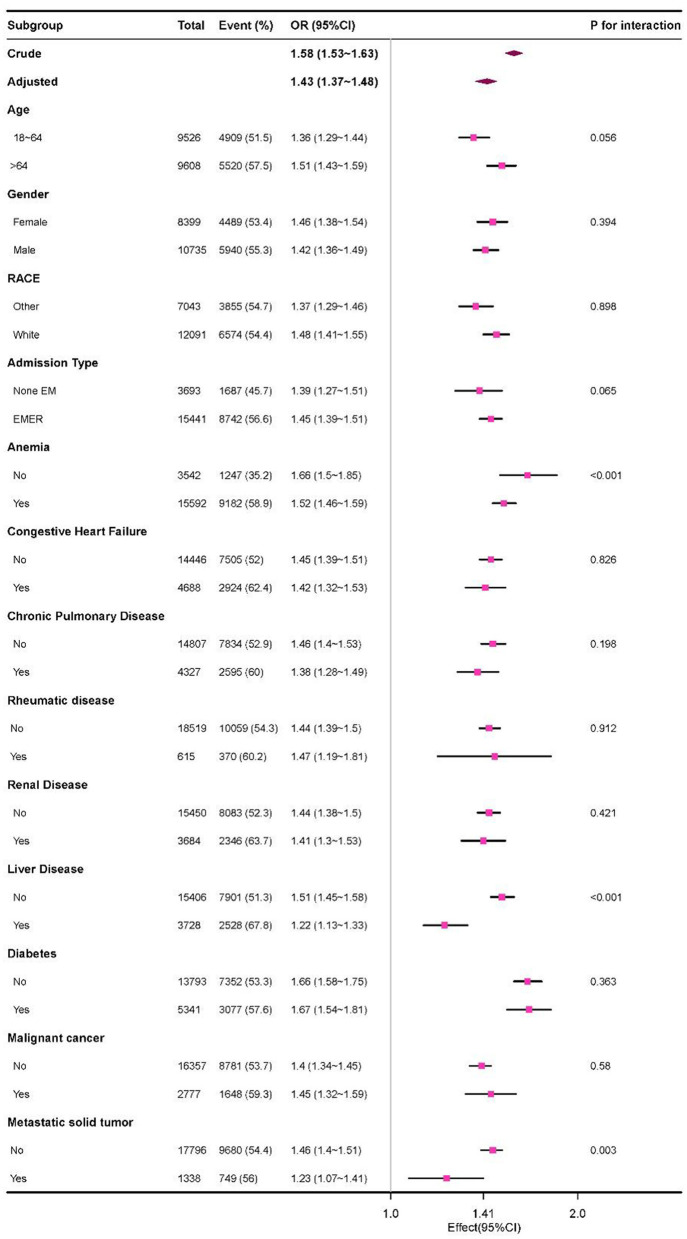
Effect size of HAG on sepsis in each subgroup.

The subgroup analysis revealed a positive correlation between HAG and sepsis (Figure 3). Although most subgroups showed no significant interactions, statistically significant interactions were observed between HAG and anemia, liver disease, and metastatic solid tumor in predicting sepsis (*P* < 0.05). However, these interactions did not compromise the stability of our main findings. Additionally, we compared the results before and after multiple imputation and found that the results remained consistent and stable (Table 2). Critically, validation in the independent MICU cohort demonstrated consistent replication of the core HAG–sepsis association. All sensitivity analyses supported our main finding that a higher HAG level was associated with an elevated risk of sepsis.

## Discussion

Our findings indicate that the HAG is significantly associated with sepsis risk in ICU patients, and this association remains consistent across common clinical complications. Compared with conventional biomarkers—procalcitonin (PCT), C-reactive protein (CRP), and lactate—the HAG demonstrates more stable performance. These traditional markers frequently show variable diagnostic accuracy due to non-infectious inflammation, delayed kinetics, or cost limitations in resource-constrained settings (4, 5). Unlike dynamic biomarkers requiring serial measurements, the HAG provides an immediate assessment upon admission.

Plasma albumin, which is essential for maintaining colloid osmotic pressure, relies on endothelial glycocalyx integrity for vascular retention (16, 17). During sepsis, inflammatory mediators (e.g., TNF-α), oxidative stress, and enzymes (e.g., matrix metalloproteinases [MMPs], heparanase-1, hyaluronidases, and neuraminidases) degrade the glycocalyx, increasing vascular permeability and promoting albumin leakage (18, 19). Concurrently, fluid shifts induce relative hemoconcentration, elevating the hematocrit. This dual mechanism—reduced albumin and elevated hematocrit—underlies the elevation of HAG in sepsis. Conditions altering baseline physiology (e.g., anemia with chronically low hematocrit, liver disease with impaired albumin synthesis, or malignancy-associated glycocalyx disruption) may attenuate the HAG–sepsis association, as observed in the subgroup analyses; however, these context-dependent variations do not compromise the robustness of the primary association.

This study identifies a distinct S-shaped non-linear association between the hematocrit–albumin gap (HAG) and sepsis risk—a pattern with dual significance. Statistically, it delineates three phases of the risk trajectory: a minimal variation at low HAG values (< −9.162), pronounced escalation across the mid-range (−9.162 to 17.27), and attenuated progression at high values (>17.27). These estimates reflect localized slope effects within predefined intervals and should not be interpreted as causal or clinically actionable thresholds. Conceptually, this non-linearity serves two critical purposes: (1) it cautions against the linear modeling of the HAG in sepsis risk assessment, as such simplification would obscure its true association pattern and introduce estimation bias; and (2) it provides a refined statistical framework for future predictive algorithm development, advocating for the incorporation of the HAG via flexible non-linear terms (e.g., restricted cubic splines) to preserve analytical fidelity.

Within established sepsis screening frameworks, the HAG serves as a pragmatic complementary metric rather than as a substitute for existing tools. SIRS criteria—requiring ≥2 abnormalities in temperature, heart rate, respiratory rate, or white blood cell count—demonstrate high sensitivity but critically low specificity in ICU settings due to frequent activation by non-infectious conditions (e.g., trauma, pancreatitis, postoperative states) (7). Although the qSOFA score (≥2 of altered mentation, systolic BP ≤ 100 mmHg, respiratory rate ≥22 breaths/min) improves specificity over SIRS, its sensitivity remains suboptimal (< 60%) in established ICU cohorts (8). Conversely, the full SOFA score, which is central to Sepsis-3 (1) for confirming organ dysfunction, requires serial assessments over 24 h and is impractical for rapid risk stratification at admission (7, 20). The HAG addresses this operational gap—derived solely from routinely available hematocrit and albumin values, it yields an objective, reproducible metric within minutes of admission, with minimal cost and reduced susceptibility to observer bias compared with SIRS or qSOFA/SOFA. Importantly, HAG is not intended for sepsis diagnosis but functions as an early trigger—notable HAG elevations may prompt immediate SOFA assessment and targeted infection evaluation per Sepsis-3 guidelines. Prior studies by Wang et al. (11) (elderly ICU cohort) and our earlier study (12) (culture-proven infections only) reported HAG's prognostic utility but were limited by narrow cohorts, the exclusion of culture-negative sepsis, and pre-Sepsis-3 diagnostic criteria. This multicenter validation, applying current Sepsis-3 definitions across diverse adult ICU populations, extends the evidence base and supports the HAG's role as a scalable adjunct for efficient risk stratification.

Despite its significant insights, this study faces several limitations: (1) While the institutional validation cohort strengthens reproducibility within a distinct healthcare context, generalizability to global populations requires prospective multicenter validation. (2) Due to substantial missing data on height within the MIMIC database, we used weight as a substitute for body mass index (BMI), which could lead to inaccuracies in assessing an individual's nutritional status. (3) The high exclusion rate due to missing hematocrit/albumin values warrants caution. Patients with available laboratory tests may differ systematically from excluded patients (e.g., severity of illness, admission urgency), potentially affecting cohort representativeness despite imputation. Future prospective studies should prioritize complete biomarker capture at admission. (4) The HAG was assessed solely at ICU admission, precluding the evaluation of dynamic changes during the ICU stay (e.g., fluid therapy effects) that may influence risk trajectories. (5) Despite comprehensive adjustment, unmeasured confounders (e.g., pre-admission comorbidities, socioeconomic factors) could affect the observed associations. (6) As this is an observational study, causality cannot be inferred. Future studies should evaluate the HAG's diagnostic and predictive performance in prospective cohorts using time-dependent ROC analysis and integrate the HAG into multivariable sepsis prediction models to assess its incremental value.

## Conclusion

This study demonstrated an S-shaped association between the HAG and sepsis. Notably, the risk intensifies when this difference falls between −9.162 and 17.27. While the HAG calculation is simple and rapid, its diagnostic utility requires prospective validation.

## Data Availability

The raw data supporting the conclusions of this article will be made available by the authors, without undue reservation.

## References

[1] Singer M Deutschman CS Seymour CW Shankar-Hari M Annane D Bauer M . The third international consensus definitions for sepsis and septic shock (Sepsis-3). JAMA. (2016) 315:801. doi: 10.1001/jama.2016.0287 26903338 PMC4968574

[2] Fleischmann C Scherag A Adhikari NKJ Hartog CS Tsaganos T Schlattmann P . Assessment of global incidence and mortality of hospital-treated sepsis. Current estimates and limitations. Am J Respir Crit Care Med. (2016) 193:259–72. doi: 10.1164/rccm.201504-0781OC 26414292

[3] Evans L Rhodes A Alhazzani W Antonelli M Coopersmith CM French C . Surviving sepsis campaign: international guidelines for management of sepsis and septic shock 2021. Intensive Care Med. (2021) 47:1181–247. 34599691 10.1007/s00134-021-06506-y PMC8486643

[4] Van Engelen TSR Wiersinga WJ Scicluna BP Van Der Poll T. Biomarkers in sepsis. Crit Care Clin. (2018) 34:139–52. doi: 10.1016/j.ccc.2017.08.010 29149935

[5] Cha K Choi SP Kim SH Oh SH. Prognostic value of ambulation ability with albumin and C-reactive protein to predict 28-day mortality in elderly sepsis patients: a retrospective multicentre registry-based study. BMC Geriatr. (2022) 22:661. doi: 10.1186/s12877-022-03339-2 35962331 PMC9373310

[6] Lee S Song J Park DW Seok H Ahn S Kim J . Diagnostic and prognostic value of presepsin and procalcitonin in non-infectious organ failure, sepsis, and septic shock: a prospective observational study according to the Sepsis-3 definitions. BMC Infect Dis. (2022) 22:8. doi: 10.1186/s12879-021-07012-8 34983420 PMC8725484

[7] Seymour CW Liu VX Iwashyna TJ Brunkhorst FM Rea TD Scherag A . Assessment of Clinical criteria for sepsis: for the third international consensus definitions for sepsis and septic shock (Sepsis-3). JAMA. (2016) 315:762–74. doi: 10.1001/jama.2016.0288 26903335 PMC5433435

[8] Fernando SM Tran A Taljaard M Cheng W Rochwerg B Seely AJE . Prognostic accuracy of the quick sequential organ failure assessment for mortality in patients with suspected infection: a systematic review and meta-analysis. Ann Intern Med. (2018) 168:266–75. doi: 10.7326/M17-2820 29404582

[9] Anand V Zhang Z Kadri SS Klompas M Rhee C CDC Prevention Epicenters Program. Epidemiology of quick sequential organ failure assessment criteria in undifferentiated patients and association with suspected infection and sepsis. Chest. (2019) 156:289–97. doi: 10.1016/j.chest.2019.03.032 30978329 PMC6859245

[10] Vincent JL de Mendonça A Cantraine F Moreno R Takala J Suter PM . Use of the SOFA score to assess the incidence of organ dysfunction/failure in intensive care units: results of a multicenter, prospective study. Working group on “sepsis-related problems” of the European Society of Intensive Care Medicine. Crit Care Med. (1998) 26:1793–800. doi: 10.1097/00003246-199811000-00016 9824069

[11] Wang Z Zhang L Li S Xu F Han D Wang H . The relationship between hematocrit and serum albumin levels difference and mortality in elderly sepsis patients in intensive care units—a retrospective study based on two large database. BMC Infect Dis. (2022) 22:629. doi: 10.1186/s12879-022-07609-7 35850582 PMC9295343

[12] Dai D-M Wang D Hu D Wan W-L Su Y Yang J-L . Difference in hematocrit and plasma albumin levels as an additional biomarker in the diagnosis of infectious disease. Arch Med Sci. (2020) 16:522–30. doi: 10.5114/aoms.2019.86898 32399098 PMC7212220

[13] Cai S Wang Q Chen C Guo C Zheng L Yuan M. Association between blood urea nitrogen to serum albumin ratio and in-hospital mortality of patients with sepsis in intensive care: a retrospective analysis of the fourth-generation Medical Information Mart for Intensive Care database. Front Nutr. (2022) 9:967332. doi: 10.3389/fnut.2022.967332 36407534 PMC9672517

[14] Xu W Huo J Cheng G Fu J Huang X Feng J . Association between different concentrations of human serum albumin and 28-day mortality in intensive care patients with sepsis: a propensity score matching analysis. Front Pharmacol. (2022) 13:1037893. doi: 10.3389/fphar.2022.1037893 36578542 PMC9792095

[15] Yang J Li Y Liu Q Li L Feng A Wang T . Brief introduction of medical database and data mining technology in big data era. J Evid Based Med. (2020) 13:57–69. doi: 10.1111/jebm.12373 32086994 PMC7065247

[16] Vercueil A Grocott MPW Mythen MG. Physiology, pharmacology, and rationale for colloid administration for the maintenance of effective hemodynamic stability in critically Ill patients. Transfus Med Rev. (2005) 19:93–109. doi: 10.1016/j.tmrv.2004.11.006 15852239

[17] Foote CA Soares RN Ramirez-Perez FI Ghiarone T Aroor A Manrique-Acevedo C . Endothelial glycocalyx. Compr Physiol. (2022) 12:3781–811. doi: 10.1002/cphy.c210029 35997082 PMC10214841

[18] Schmidt EP Yang Y Janssen WJ Gandjeva A Perez MJ Barthel L . The pulmonary endothelial glycocalyx regulates neutrophil adhesion and lung injury during experimental sepsis. Nat Med. (2012) 18:1217–23. doi: 10.1038/nm.2843 22820644 PMC3723751

[19] Goligorsky MS Sun D. Glycocalyx in endotoxemia and sepsis. Am. J. Pathol. (2020) 190:791–8. doi: 10.1016/j.ajpath.2019.06.017 32035882 PMC7180514

[20] Shankar-Hari M Phillips GS Levy ML Seymour CW Liu VX Deutschman CS . Developing a new definition and assessing new clinical criteria for septic shock: for the third international consensus definitions for sepsis and septic shock (Sepsis-3). JAMA. (2016) 315:775–87. doi: 10.1001/jama.2016.0289 26903336 PMC4910392

