# Supplementary material for: The association between hematocrit–albumin gap and sepsis risk in ICU patients: a multicenter cross-sectional study
Source: Front Med (Lausanne). 2026 Apr 17;13:1747343. doi: 10.3389/fmed.2026.1747343 (PMC13133070; doi:10.3389/fmed.2026.1747343)
Supplement: Supplementary file 1 [file Table_1.docx]

Additional file 1a: Association of covariates and sepsis risk in the derivation cohort.

| Variable | OR (95%CI) | P-value |
| --- | --- | --- |
| HAG | 1.58 (1.53~1.63) | <0.001 |
| Age | 1.01 (1.01~1.01) | <0.001 |
| Gender |  |  |
| Male | 1.08 (1.02~1.14) | 0.009 |
| RACE |  |  |
| WHITE | 0.99 (0.93~1.05) | 0.626 |
| Weight | 1 (1~1) | <0.001 |
| Admission Type |  |  |
| EMER | 1.55 (1.44~1.67) | <0.001 |
| Vital signs |  |  |
| Temperature | 1.72 (1.65~1.79) | <0.001 |
| Heart rate | 1.02 (1.01~1.02) | <0.001 |
| MBP | 0.96 (0.96~0.96) | <0.001 |
| RR | 1.04 (1.04~1.05) | <0.001 |
| Laboratory Metrics |  |  |
| WBC | 1.04 (1.04~1.05) | <0.001 |
| Glucose | 1 (1~1) | <0.001 |
| Platelets | 1 (1~1) | <0.001 |
| ALT | 1 (1~1) | <0.001 |
| AST | 1 (1~1) | <0.001 |
| ALP | 1 (1~1) | <0.001 |
| Creatinine | 1.21 (1.18~1.23) | <0.001 |
| BUN | 1.02 (1.02~1.02) | <0.001 |
| Lac | 1.16 (1.14~1.17) | <0.001 |
| AG | 1.05 (1.04~1.05) | <0.001 |
| Bicarbonate | 0.94 (0.93~0.94) | <0.001 |
| Medical history |  |  |
| Congestive Heart Failure | 1.53 (1.43~1.64) | <0.001 |
| Chronic Pulmonary Disease | 1.33 (1.24~1.43) | <0.001 |
| Rheumatic Disease | 1.27 (1.08~1.5) | 0.004 |
| Liver Disease | 2.01 (1.87~2.18) | <0.001 |
| Renal Disease | 1.6 (1.48~1.72) | <0.001 |
| Diabetes | 1.19 (1.12~1.27) | <0.001 |
| Malignant cancer | 1.26 (1.16~1.37) | <0.001 |
| Metastatic solid tumor | 1.07 (0.95~1.19) | 0.262 |

Abbreviations: HAG, hematocrit-albumin gap; EM, emergency; MBP, mean blood pressure; RR, Respiration rate, WBC, white blood cell; ALT, alanine aminotransferase; AST, aspartate aminotransferase; ALP, alkaline Phosphatase; BUN, blood urea nitrogen; Lac, lactate; AG, anion gap.

Additional file 1b: Association of covariates and sepsis risk in the validation cohort.

| Variable | OR (95%CI) | P-value |
| --- | --- | --- |
| HAG | 1.1 (1.08~1.13) | <0.001 |
| Age | 1.02 (1.01~1.03) | 0.003 |
| Gender |  |  |
| Male | 0.64 (0.46~0.9) | 0.011 |
| Weight | 1.03 (1.01~1.04) | <0.001 |
| Admission Type |  |  |
| EMER | 0.99 (0.71~1.39) | 0.967 |
| Vital signs |  |  |
| Temperature | 1.29 (1.08~1.54) | 0.005 |
| Heart rate | 1.01 (1.01~1.02) | <0.001 |
| MBP | 0.99 (0.99~1) | 0.052 |
| RR | 1.04 (1.02~1.07) | 0.001 |
| Laboratory Metrics |  |  |
| WBC | 1 (0.99~1.01) | 0.5 |
| Glucose | 1.02 (0.99~1.05) | 0.167 |
| Platelets | 1 (0.99~1) | <0.001 |
| ALT | 1 (1~1) | 0.979 |
| AST | 1 (1~1) | 0.539 |
| ALP | 1 (1~1.01) | 0.001 |
| Creatinine | 1 (1~1) | 0.68 |
| BUN | 1.02 (1~1.03) | 0.025 |
| Lac | 1.1 (1.04~1.16) | 0.001 |
| AG | 1.04 (1.01~1.06) | 0.009 |
| Bicarbonate | 0.94 (0.92~0.97) | <0.001 |
| Medical history |  |  |
| Congestive Heart Failure | 0.91 (0.61~1.35) | 0.623 |
| Chronic Pulmonary Disease | 0.98 (0.71~1.37) | 0.917 |
| Rheumatic Disease | 0.88 (0.57~1.36) | 0.57 |
| Liver Disease | 1 (0.58~1.71) | 0.999 |
| Renal Disease | 1.13 (0.73~1.75) | 0.593 |
| Diabetes | 1.04 (0.74~1.48) | 0.806 |
| Malignant cancer | 1.36 (0.83~2.24) | 0.226 |
| Metastatic solid tumor | 0.8 (0.32~2.02) | 0.636 |

Abbreviations: HAG, hematocrit-albumin gap; EM, emergency; MBP, mean blood pressure; RR, Respiration rate, WBC, white blood cell; ALT, alanine aminotransferase; AST, aspartate aminotransferase; ALP, alkaline Phosphatase; BUN, blood urea nitrogen; Lac, lactate; AG, anion gap.
